# Cohort Profile: The Flu Watch Study

**DOI:** 10.1093/ije/dyv370

**Published:** 2016-03-03

**Authors:** Ellen B Fragaszy, Charlotte Warren-Gash, Lili Wang, Andrew Copas, Oliver Dukes, W John Edmunds, Nilu Goonetilleke, Gabrielle Harvey, Anne M Johnson, Jana Kovar, Megan SC Lim, Andrew McMichael, Elizabeth RC Millett, Irwin Nazareth, Jonathan S Nguyen-Van-Tam, Faiza Tabassum, John M Watson, Fatima Wurie, Maria Zambon, Andrew C Hayward, on behalf of the Flu Watch Group

**Affiliations:** 1 Institute of Health Informatics, University College London, London, UK,; 2 London School of Hygiene & Tropical Medicine, London, UK,; 3 Weatherall Institute of Molecular Medicine, University of Oxford, Oxford, UK,; 4 Research Department of Infection & Population Health, University College London, London, UK,; 5 Department of Microbiology and Immunology, University of North Carolina at Chapel Hill, Chapel Hill, CA, USA,; 6 Burnet Institute, Centre for Population Health, Melbourne, VIC, Australia,; 7 Nuffield Department of Medicine, University of Oxford, Oxford, UK,; 8 Research Department of Primary Care and Population Health, University College London, London, UK,; 9 Division of Epidemiology and Public Health, University of Nottingham, Nottingham, UK,; 10 Chief Medical Officer's Private Office, Department of Health, London, UK and; 11 Public Health England Respiratory Virus Unit, Colindale, UK

## Why was the cohort set up?

Influenza is a common, highly contagious respiratory virus which infects all age groups, causing a range of outcomes from asymptomatic infection and mild respiratory disease to severe respiratory disease and death.^1^ If infected, the adaptive immune system produces a humoral (antibody) and cell-mediated (T cell) immune response to fight the infection.^2^ Influenza viruses continually evolve through antigenic drift, resulting in slightly different ‘seasonal’ influenza strains circulating each year. Population-level antibody immunity to these seasonal viruses builds up over time, so in any given season only a proportion of the population is susceptible to the circulating strains. Occasionally, influenza A viruses evolve rapidly through antigenic shift by swapping genes with influenza viruses usually circulating in animals. This process creates an immunologically distinct virus to which the population may have little to no antibody immunity. The virus can result in a pandemic if a large portion of the population is susceptible and the virus is easily spread.^1^

International influenza surveillance is typically based upon cases seeking medical care.^3–5^ However, this focus greatly underestimates the true community burden of seasonal influenza: the majority of cases are mild and self-limiting, with asymptomatic infections accounting for 25% to 75% of all infections.^6^^,^^7^ Effective influenza control requires knowledge of disease burden and factors affecting influenza transmission. Existing parameters for mathematical models of influenza interventions are largely derived from household cohort studies conducted in the USA between 1948 and 1981.^8–10^ Since then there have been profound social changes affecting population contact and mixing patterns that are likely to impact on influenza transmission. These changes include more women working, more children attending day care, more commuting and international travel and increased vaccine coverage. Evolutionary changes to circulating viruses may affect transmission dynamics, patterns of clinical illness and the adaptive immune responses elicited.^1^^,^^11^ Rapid advances in laboratory methods have also occurred, providing unique opportunities to investigate immune correlates, both humoral and T cell based, with influenza infection rates and disease severity.^11^^,^^12^

The initial Flu Watch cohort, funded by the UK Medical Research Council (MRC), began in 2006 as a collaboration between epidemiologists at the Centre for Infectious Disease Epidemiology at University College London (UCL), virologists and mathematical modellers from the Health Protection Agency (HPA, now Public Health England), immunologists at the MRC Human Immunology Unit at Oxford University and the MRC General Practice Research Framework (GPRF). It aimed to estimate community burden of influenza and influenza-like illness, generate up-to-date knowledge of demographic, social and behavioural factors affecting influenza transmission, measure antibody and T cell immune responses to influenza and to use knowledge generated to inform modelling parameters. In addition, a pandemic preparedness cohort was envisioned, in which participants already familiar with the study consented to be re-contacted in the event of a pandemic, to allow rapid redeployment of the study.

When the 2009 influenza AH1N1 pandemic arose, further funding was secured jointly from the MRC and Wellcome Trust, allowing continued follow-up and an expansion in cohort size. New collaborators for this phase included the MRC Centre for Outbreak Analysis and Modelling, the Wellcome Sanger Institute, the Primary Care Research Network and additional epidemiology and public health experts from the HPA. Additional study aims were to inform the national and international response to the current and future pandemics. Specific objectives were to examine clinical profiles of illness, estimate population infection denominators and case fatality risk, describe epidemiological characteristics of the infection in real-time, monitor changes in population behaviour, and investigate access to services, attitudes to and uptake of antivirals and vaccine, and immunity to infection in order to inform vaccination policy and development. During the pandemic, Flu Watch also provided control data and samples for studies of severe influenza (MOSAIC) and studies of influenza infection risk in people working with pigs (COSI).^13^^,^^14^

## Who is in the cohort?

Households were recruited from registers of 146 volunteer general practices (GP) across England, who formed part of the MRC GPRF or (from the 2009 pandemic onwards) the Primary Care Research Network. Participants were selected from GP lists by computer-based random number generation. GPs sent invitation letters inviting the randomly selected person and their household to participate. Although it was recognized that this would bias invitations towards larger households, such as those with children, this was accepted as the role of children in influenza transmission was an important research question. Weighting by the inverse of household size in analyses was planned to account for this sampling design.

To be eligible to participate, the whole household had to agree to take part in follow-up over the coming winter, with adults aged ≥ 16 years agreeing to have blood samples taken. Exclusion criteria included household size > 6 people, individuals with terminal illness, severe mental illness or incapacity and heavy involvement in other ongoing research. GPs reviewed invitation lists and removed anyone meeting these criteria, before sending letters. Cohorts were recruited to allow follow-up of participants over six influenza seasons—the 2006/07, 2007/08 and 2008/09 periods of seasonal influenza circulation, the summer and winter waves of the 2009 pandemic and the first post-pandemic season 2010/11. From season 3 (2008/09) onwards, previous participants were invited to take part again.

In season 1, invitation letters were sent to 2300 households from 42 practices, and 602 individuals from 243 households agreed to participate. In subsequent seasons the response rate was not monitored as practices (rather than the university study team) sent the invitation letters and not all returned data on numbers sent. Compared with the English population, young adults, non-White ethnic groups, people living in socially deprived areas and those living in the North of England, West Midlands and London were under-represented in the Flu Watch cohort (Table 1).

**Table 1.** Baseline characteristics of responders by season compared with national averages

	National	Nov 2006 to Mar 2007 Season 1	Nov 2007 to Mar 2008 Season 2	Nov 2008 to Mar 2009 Season 3	May 2009 to Sep 2009 Season 4	Oct 2009 to Feb 2010 Season 5	Nov 2010 to Mar 2011 Season 6
**GP practices/ households/ persons (*n*)**		42/243/602	43/310/779	37/309/729	41/332/797	127/1460/3552	51/361/901
**Age group**							
0 to 4 years	6%	38 (6.31%)	42 (5.39%)	37 (5.08%)	36 (4.52%)	179 (5.04%)	45 (4.99%)
5 to 15	11%	87 (14.45%)	110 (14.12%)	99 (13.58%)	109 (13.68%)	501 (14.10%)	131 (14.54%)
16 to 44	42%	151 (25.08%)	258 (33.12%)	172 (23.59%)	192 (24.09%)	848 (23.87%)	206 (22.86%)
45 to 64	25%	203 (33.72%)	272 (34.92%)	267 (36.63%)	293 (36.76%)	1225 (34.49%)	344 (38.18%)
65+	16%	123 (20.43%)	97 (12.45%)	154 (21.12%)	167 (20.95%)	799 (22.49%)	175 (19.42%)
**Gender**							
Male	49%	281 (46.68%)	366 (46.98%)	340 (46.64%)	377 (47.30%)	1740 (48.99%)	455 (50.50%)
Female	51%	321 (53.32%)	413 (53.02%)	389 (53.36%)	420 (52.70%)	1812 (51.01%)	446 (49.50%)
**Region**							
North	28%	99 (16.45%)	89 (11.42%)	100 (13.72%)	106 (13.30%)	320 (9.01%)	115 (12.76%)
West Midlands	11%	42 (6.98%)	96 (12.32%)	46 (6.31%)	53 (6.65%)	179 (5.04%)	53 (5.88%)
East & East Midlands	20%	122 (20.27%)	120 (15.40%)	124 (17.01%)	118 (14.81%)	1456 (40.99%)	321 (35.63%)
London	15%	28 (4.65%)	77 (9.88%)	26 (3.57%)	28 (3.51%)	270 (7.60%)	65 (7.21%)
South East	16%	100 (16.61%)	117 (15.02%)	107 (14.68%)	155 (19.45%)	319 (8.98%)	110 (12.21%)
South West	10%	211 (35.05%)	280 (35.94%)	326 (44.72%)	337 (42.28%)	1008 (28.38%)	237 (26.30%)
**Vaccine**							
Vaccinated^a^		115 (19.10%)	130 (16.69%)	169 (23.18%)	0 (0%)	157 (4.42%)	186 (20.64%)
Unvaccinated		462 (76.74%)	632 (81.13%)	527 (72.29%)	797 (100%)	3159 (88.94%)	715 (79.36%)
Unknown		25 (4.15%)	17 (2.18%)	33 (4.53%)	0 (0%)	236 (6.64%)	0 (0%)
**Index of Multiple Deprivation quintile**							
1 (most deprived)	20%	37 (6.15%)	39 (5.01%)	28 (3.84%)	18 (2.26%)	98 (2.76%)	29 (3.22%)
2	20%	88 (14.62%)	126 (16.17%)	91 (12.48%)	62 (7.78%)	310 (8.73%)	82 (9.10%)
3	20%	164 (27.24%)	235 (30.17%)	238 (32.65%)	146 (18.32%)	915 (25.76%)	221 (24.53%)
4	20%	162 (26.91%)	250 (32.09%)	187 (25.65%)	146 (18.32%)	938 (26.41%)	280 (31.08%)
5 (least deprived)	20%	151 (25.08%)	129 (16.56%)	185 (25.38%)	425 (53.32%)	1291 (56.35%)	289 (32.08%)
**Ethnicity** White	75%	557 (97.89%)	733 (95.44%)	666 (99.11%)	730 (99.05%)	3306 (97.70%)	846 (97.80%)
Non-White	25%	5 (2.11%)	3 (4.56%)	6 (0.89%)	7 (0.95%)	78 (2.30%)	19 (2.20%)

*Vaccinated for that influenza season (before or during follow-up).

## How often have they been followed up?

### The basic cohort design

#### Baseline/pre-season phase

A baseline visit was made to the household at enrolment, during which a research nurse collected blood samples for serological and T cell analysis from all adults aged 16 years or older. Blood sampling was optional for those aged 5–15 years and not done in those under 5 years of age. Visits occurred in the evenings, as bloods had to be couriered overnight to Oxford for early morning analysis of T cells. The serum samples collected we recentrifuged, frozen and later batch-tested for influenza antibodies by the HPA. Nurses assisted families with a series of laptop-based surveys collecting information on basic demographics, health and chronic illness, respiratory hygiene, household structure and relationships, accommodation, contacts and activities. Households received participant packs containing paper illness diaries, thermometers and nasal swab kits including instructions on their use and the viral transport medium to be stored in the refrigerator.

#### Active follow-up during influenza season

In order to obtain reliable measures of the number of illnesses, we actively contacted participants every week with automated telephone calls to assess the presence or absence of respiratory illness in each household member. For each respiratory illness, participants were reminded to fill in a prospective paper illness diary. These collected information on illness onset date, temperature and presence and severity of symptoms such as feeling feverish, headache, muscle aches, cough and sore throat. Diaries also collected data on contact patterns and activities before and during illness. Participants took a nasal swab on day 2 of any respiratory illness for polymerase chain reaction (PCR) analysis of influenza, respiratory syncytial virus (RSV), human metapneumovirus (hMPV), rhinovirus, coronavirus, adenovirus and parainfluenzavirus. During the first season, swabbing was limited to periods of influenza circulation. The Sanger Institute genetically sequenced some of the viral isolates from the summer and winter waves of the pandemic (seasons 4–5).

In addition, all participants completed one-off activity and contact paper diaries on at least 1 pre-determined weekday and 1 weekend day during the active follow-up period. These diaries collected information on where participants were (i.e. at home, at work etc.), whether they had contact with crowds and the number, duration and age groups of personal contacts throughout the day.

#### Post-season phase

At the end of follow-up, nurses made a final household visit to take a follow-up blood sample (for paired serology) and assist participants with an exit survey. Nurses also checked participants’ medical records for information on chronic illnesses, influenza and pneumococcal vaccinations, prescriptions, GP consultations, hospitalizations and deaths.

### Evolution of data collection

The cohort evolved over time to maximize system reliability, minimize the number of data sources and allow increased recruitment during the pandemic. In season 3 we offered participants the option of moving from paper illness diaries with weekly automated phone calls to weekly emailed surveys with or without optional SMS reminders. For the pandemic and post-pandemic cohort, most surveys moved to a custom-built website for self-completion. In order to achieve real-time monitoring of illnesses during the pandemic, participants were emailed a link to a retrospective online weekly survey and provided with laminated wipe-clean charts at home to record daily symptoms as a memory aid.

In season 3 there were additional one-off surveys collecting data on indoor and outdoor temperature and humidity, travel patterns and non-response to weekly surveys. During seasons 5 and 6 we added questions to existing surveys on attitudes towards influenza vaccination and antivirals. In season 6 we included quality of life questions.^15^

### Evolution of cohort design

The cohort design evolved with the emergence of the novel H1N1 pandemic strain during season 3.We continued active follow-up through the UK summer wave of the pandemic (season 4). For the UK winter wave of the pandemic (season 5), the study split into three separate cohorts: T cell (comprising both previous and newly recruited participants), Serology and Virology (both comprising new participants). For the T cell cohort, continuing participants used the spring blood sample from season 3 as a baseline sample. They also gave a pre-vaccination blood sample to allow distinction of antibody rises caused by infection rather than vaccination. This was particularly important for the winter wave of the pandemic, as we anticipated widespread vaccination. The Serology cohort was identical but lacked T cell samples. For the Virology cohort, no blood samples were taken. This allowed for rapid recruitment of a large number of participants (*n* = 1778) to increase the accuracy of weekly estimates of illness rates during the pandemic, with minimal nurse time required. All nasal swabs were tested for influenza A and B, RSV and hMPV but, due to the large number of samples generated during the pandemic, only a selection in seasons 5 and 6 were tested for other viruses.

### Loss to follow-up and missing data

**Figure 1:**
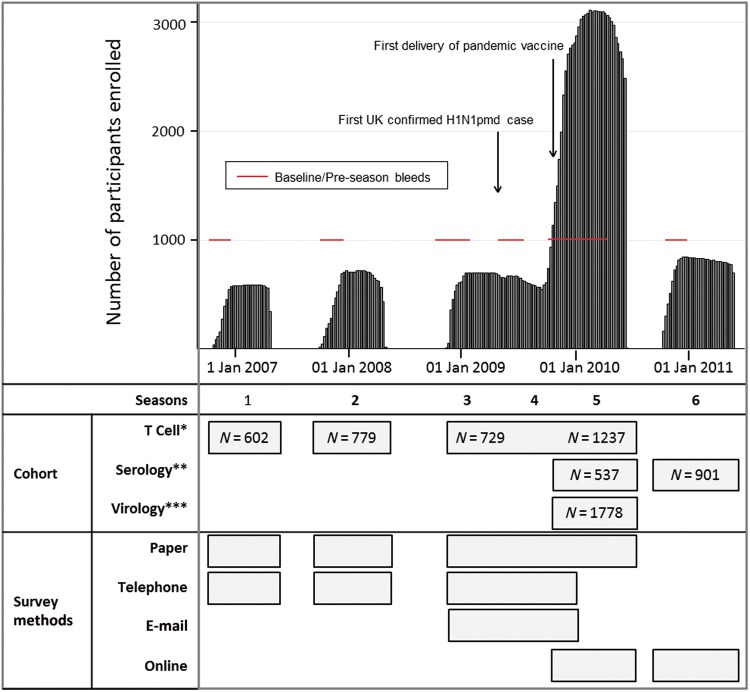
Number of enrolled participants, baseline/pre-season bleed periods and different cohorts and data collection methods over time. ‘Survey Methods’ boxes used to indicate which methods were used to follow up participants in each season *T cell cohorts included T cell, serological and virological (PCR) measurements. ** Serology cohorts included serological and virological (PCR) measurements. *** Virology cohort only included virological (PCR) measurements.

Retention of enrolled participants throughout the cohorts was good. Figure 1 displays the number of enrolled participants each week, with arrows pointing out the staggered starts and exits of the cohorts along with other important dates. Loss to follow-up came in two main varieties: non-response to weekly contact and loss to follow-up for paired blood samples.

We obtained weekly responses from 87.3% of follow-up weeks overall, which increased to 88.4% if we exclude periods when there were technical difficulties with our automated phone calls (1 week in season 1 and 4 weeks in season 2). Response completeness generally increased after the introduction of email and online surveys in season 3 (Table 2). Only 12.4% of households were classified as poor responders (responding to < 70% of follow-up weeks). Poor response appeared to be more common as deprivation increased.

**Table 2.** Characteristics of non-responding households (i.e. households with ≥ 30% missing weeks)

Household characteristics	Good responders		Poor responders		Total
	(< 30% missing weeks)		(≥ 30% missing weeks)		
	*N*	%	*N*	%	*N*
**Overall**	2640	87.6	372	12.4	3012
**Season**					
Nov 2006 to Mar 2007 (1)	199	81.9	44	18.1	243
Nov 2007 to Mar 2008 (2)	202	65.8	105	34.2	307
Nov 2008 to Mar 2009 (3)	287	92.9	22	7.1	309
May 2009 to Sep 2010 (4)	246	74.1	86	25.9*	332
Oct 2009 to Feb 2010 (5)	1370	93.8	90	6.2	1460
Nov 2010 to Mar 2011 (6)	336	93.1	25	6.9	361
**Social class**					
Managerial and professional	712	87.6	101	12.4	813
Intermediate occupations	362	87.9	50	12.1	412
Small employers and own-account workers	209	85.3	36	14.7	245
Lower supervisory and technical occupations	111	84.1	21	15.9	132
Semi-routine and routine occupations	441	86.5	69	13.5	510
Retired	497	94	32	6	529
Student	109	84.5	20	15.5	129
missing	199	82.2	43	17.8	242
**Index of Multiple Deprivation quintile**					
1 (most deprived)	85	81	20	19	105
2	255	84.7	46	15.3	301
3	704	86.9	106	13.1	810
4	732	89.6	85	10.4	817
5 (least deprived)	864	88.3	115	11.7	979
**Rural/urban**					
Urban > 10k	1505	86.7	230	13.3	1735
Town and fringe	373	90.3	40	9.7	413
Village, hamlet and isolated dwellings	643	89.9	72	10.1	715
Missing	119	79.9	30	20.1	149
**Household size**					
1	354	84.5	65	15.5	419
2	1405	89.7	162	10.3	1567
3	344	85.1	60	14.9	404
4	407	87.3	59	12.7	466
5	109	84.5	20	15.5	129
6	21	77.8	6	22.2	27
**Number of children in the household**					
0	1932	89.1	236	10.9	2168
1	247	81.8	55	18.2	302
2	360	85.1	63	14.9	423
3	83	86.5	13	13.5	96
4	18	78.3	5	21.7	23
**Region**					
North	305	87.9	42	12.1	347
West Midlands	164	84.1	31	15.9	195
East and East Midlands	828	90.5	87	9.5	915
London	164	84.5	30	15.5	194
South East	314	83.5	62	16.5	376
South West	865	87.8	120	12.2	985

^a^We believe the poor response in this season may be due to summer holidays.

We obtained paired blood samples from 80% of participants required to provide them and from 27% of participants aged 15 and under, for whom blood samples were optional (Table 3).

**Table 3.** Characteristics of Participants with and without missing blood samples by whether or not those blood samples were required or optional

Individual characteristics	Participants with Mandatory Bloods					Participants with Optional Bloods				
	Paired Bloods		Missing Blood		Total	Paired Bloods		Missing Blood		Total
	*N*	%	*N*	%	*N*	*N*	%	*N*	%	*N*
**Overall**	3114	80.5	754	19.5	3868	181	27.0	489	73.0	670
**Season**										
Nov 2006 to Mar 2007 (1)	422	88.5	55	11.5	477	31	35.6	56	64.4	87
Nov 2007 to Mar 2008 (2)	503	80.2	124	19.8	627	27	24.5	83	75.5	110
Nov 2008 to Mar 2009 (3)	489	82.5	104	17.5	593	23	23.2	76	76.8	99
Oct 2009 to Feb 2010 (5)	1120	77.5	326	22.5	1446	70	28.8	173	71.2	243
Nov 2010 to Mar 2011 (6)	580	80.0	145	20.0	725	30	22.9	101	77.1	131
**Gender**										
Male	1441	79.8	363	20.1	1804	95	27.7	248	72.3	343
Female	1673	81.0	391	18.9	2064	86	26.3	241	73.7	327
**Age group**										
Age 5 to 15 years	n/a		n/a			181	27.0	489	73.0	670
Age 16 to 44 years	874	74.0	307	26.0	1181	n/a		n/a		
Age 45 to 64 years	1446	82.4	309	17.6	1755	n/a		n/a		
Age 65 and over	794	85.2	138	14.8	932	n/a		n/a		
**Region**										
North	365	73.9	129	26.1	494	25	28.1	64	71.9	89
West Midlands	231	84.3	43	15.7	274	10	23.8	32	76.2	42
East & East Midlands	817	79.7	208	20.3	1025	43	23.0	144	77.0	187
London	158	84.5	29	15.5	187	13	30.2	30	69.8	43
South East	444	79.7	113	20.3	557	25	33.3	50	66.7	75
South West	1099	82.6	232	17.4	1331	65	27.8	169	72.2	234
**Vaccine**										
Vaccinated^a^	953	84.0	181	16.0	1134	14	29.8	33	70.2	47
Unvaccinated	2072	81.1	484	18.9	2554	165	27.9	427	72.1	592
Unknown	89	49.4	91	50.6	180	2	6.5	29	93.5	31
**Index of Multiple Deprivation (National quintile)**										
1 (most deprived)	110	86.6	17	13.4	127	6	20.7	23	79.3	29
2	363	84.6	66	15.4	429	21	28.4	53	71.6	74
3	893	81.8	199	18.2	1092	59	30.1	137	69.9	196
4	922	83.3	185	16.7	1107	50	27.5	132	72.5	182
5 (least deprived)	826	74.2	287	25.8	1113	45	23.8	144	76.2	189
**Ethnicity**										
White	2654	82.8	551	17.2	3205	161	29.1	392	70.9	553
Non-White	49	70.0	21	30.0	70	1	9.1	10	90.9	11
Missing	411	69.3	182	30.7	593	19	17.9	87	82.1	106
**Rural/Urban**										
Urban	1895	82.5	403	17.5	2298	116	26.3	325	73.7	441
Town and Fringe	426	82.4	91	17.6	517	23	33.3	46	66.7	69
Village, hamlet and isolated Dwellings	793	82.1	173	17.9	966	42	30.9	94	69.1	136
Missing	0	0.0	87	100.0	87	0	0.0	24	100.0	24

*Vaccinated for that influenza season (before or during follow-up).

## What has been measured?

The three main clinical outcomes were: (i) influenza-like-illness (ILI), defined as a respiratory illness with cough and/or sore throat and fever > 37.8°C;(ii) PCR-confirmed influenza illness; and (iii) influenza seroconversion, defined as a 4-fold titre rise in strain-specific antibody titres in unvaccinated individuals. Table 4 summarizes the data and biological samples collected during baseline, active follow-up and post-season phases. We additionally linked participants’ data to small area statistics such as the index of multiple deprivation and rural/urban indicators.^16^^,^^17^ Details of the T cell methodology have been described previously.^18–20^

**Table 4.** Questionnaire data and biological samples collected in three data collection periods

Phase	Data type	Measurement	Season					
			1	2	3	4	5	6
**Baseline/Pre-season**	**Self-reported surveys**	Basic demographic, socioeconomic, health, vaccination and potential risk factors for influenza	X	X	X	X	X	X
		Quality of life (EQ5D)						X
	**Blood samples**	H1N1, H3N2 and Flu B serology^a^	X	X	X			
		H1N1pdm09 serological^a^				X	X	X
		T cell analysis^b^	X	X	X		X	
**Active follow-up**	**Self-reported surveys**	Timing and characteristics of respiratory illnesses (if ill)	X	X	X	X	X	X
		Risk factors in previous week (if ill)	X	X	X			
		Time off work/education (if ill)	X	X	X	X		
		Health-seeking behaviour and medicines taken (if ill)					X	X
		Full contact and activity diaries (if ill)	X	X				
		Basic contact and activities (if ill)				X		
		Influenza vaccination that week				X	X	X
		Full contact and activity diaries (one-off survey)	X	X	X	X	X	X
		Indoor/outdoor temperature and humidity (one-off surveys)	X	X	X			
		Detailed travel survey (one-off survey)			X			
	**Self-administered nasal swabs**	RT-PCR Influenza A (H1 and H3 subtypes), influenza B, RSV and human metapneumovirus	X	X	X	X	X	X
		RT-PCR influenza A H1N1pdm09				X	X	X
		RT-PCR rhinovirus, coronavirus, adenovirus and para-influenza virus^c^	X	X	X	X	X	X
		Selected viral samples genetically sequenced	X	X	X	X	X	X
	**Blood samples****^d^**	H1N1pdm09 serology					X	
**Post-season**	**Self-reported surveys**	Changed household composition, pregnancy, vaccination, hospitalization, death and air travel	X	X	X	X	X	X
		Illness-reporting behaviour during follow-up			X		X	X
		Attitudes towards vaccination and antivirals					X	X
	**Medical records****^e^**	Chronic illness, vaccination, prescriptions, GP and hospital consultations and death	X	X	X	X	X	X
	**Blood samples**	H1N1, H3N2 and flu B serology^a^	X	X	X			
		H1N1pdm09 serology^a^					X	X
		T cell analysis^b^	X		X			
	**Saliva Samples****^f^**	Genetic analysis	X	X	X	X	X	X

^a^Haemagglutination-inhibition assay.

^b^Peripheral blood mononuclear cells (PBMC) separated, part of the sample was immediately tested against pools of peptides representing each of the virus proteins in an ex vivo IFN-γelispot assay.^18^^,^^19^ The rest of the sample was frozen down for more detailed peptide mapping studies using IFN-γelispots and/or in vitro culture and testing by intracellular cytokine staining to determine CD8/4 restriction. Post-season T cell analysis was only conducted in seasons 1 and 3.

^c^Only a selection of nasal swab samples were tested for these viruses in seasons 5 and 6.

^d^Only taken from participants in T cell and serology cohorts before influenza vaccination.

^e^Medical record checks were requested for all participants except those in the virology cohort.

^f^Saliva was collected in 2011–12 from selected participants participating from all seasons and cohorts.

## What has been found? Key findings and publications

**Figure 2:**
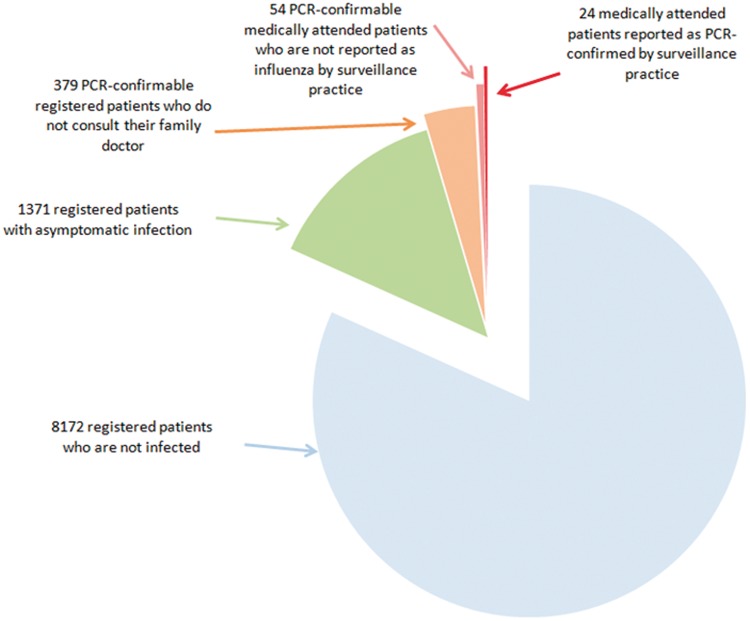
Number of expected events in a surveillance practice serving a population of 10 000: data for a typical influenza season.

Our first publication provided comprehensive national estimates of clinical and sub-clinical disease burden in the community regardless of consultations, and allowed comparison between seasonal and pandemic influenza.^2^ We found that on average, influenza infected 18% of unvaccinated people each winter and up to 75% of these infections were asymptomatic. Approximately 25% of infections were PCR confirmed and only 17% of people with PCR-confirmed disease sought medical attention; Figure 2 indicates how the primary care-based surveillance underestimated the burden of infection in the community. Results were similar between pandemic and seasonal influenza, although people infected with the 2009 pandemic strain had less severe symptoms than those infected with seasonal H3N2 strains.

Our second publication provided strong evidence that naturally occurring, cross-protective T cell immunity protects those infected with influenza against developing disease in seasonal and pandemic periods.^16^ This protection was independent of baseline antibodies and protective levels of influenza-specific T cells were found in 43% of the population. These findings help explain why such a large proportion of infections remain asymptomatic and have implications for the development of cross-protective ‘universal’ vaccines based on this response.

In order to evaluate different methods of collecting data during a pandemic, we compared prospectively collected Flu Watch data on illnesses and vaccine uptake with retrospectively collected data from the Health Survey for England.^21^ We found that retrospectively collected data underestimated disease burden but accurately estimated vaccine uptake when compared with prospectively collected data.

Current work includes an analysis of occupational exposure to pigs as a risk factor for human infection with swine and human influenza viruses; age as a predictor of T cell responses; and a comparison of serological pandemic infection rates from Flu Watch and the Health Survey for England.

## What are the main strengths and weaknesses?

Flu watch is a large community cohort study broadly representative of the population of England. It is the first modern-day household study of influenza transmission in a temperate climate, comparable to the landmark Tecumseh studies of the 1960s and 70s.^22^ A major strength is the inclusion of different household types (rather than just households with children, as in earlier studies) which allows influenza infections to be explored across the whole of society. We used highly active methods of surveillance for influenza and other respiratory viruses, exploiting a range of IT-based technologies including automated telephone surveys, e-mail, internet and text messages. Broadly similar methods of follow-up were used across six influenza seasons, allowing accurate comparisons of disease burden estimates between seasonal and pandemic influenza despite external factors (such as media reporting during the pandemic) that may have affected consultation behaviour. Robust definitions of influenza were based on a range of diagnostic methods including real-time symptom reporting, PCR and serology, allowing the emergence of the 2009 H1N1 pandemic strain to be tracked. Serological and virological data from previous pandemics are either unavailable (1918 H1N1 pandemic), from small samples sizes (1957 H2N2 pandemic)^23^ or from populations with high vaccination rates which greatly limits interpretation (1968 H3N2 pandemic).^22^ Historical data on laboratory-confirmed rates of seasonal influenza mainly come from historical community studies of families in the USA between 1948 and 1981.^10^^,^^22^^,^^24^^,^^25^ Flu Watch is a good example of collaboration between disciplines (epidemiology, immunology, virology and primary care) and partners. The study provides a rich source of data on social, behavioural and biological factors affecting influenza transmission, enabling exploration of many research questions.

Limitations include delays in obtaining funding, ethics and R&D approval across multiple sites, resulting in delayed recruitment during the pandemic and fewer participants overall. Although the initial response to invitation letters was low, it is unclear if this would bias results. Ideally, cohorts would have had pre- and post-influenza season bleeds, but recruitment periods were not perfectly streamlined with influenza seasons so adjustments for bleed timings were made during analysis. The study design and data collection methods evolved in response to experience and changing questions. Whereas this optimized and streamlined methods, it also increased complexity of data management.

## Can I get hold of the data? Where can I find out more

For further information about Flu Watch see [http://www.fluwatch.co.uk/]. Currently data are not open access but strategic collaborations are welcomed. Please address enquiries to Professor Andrew Hayward [a.hayward@ucl.ac.uk].

Flu watch profile in a Nutshell

- Flu Watch is a national prospective cohort study of influenza in English households.
- It aimed to measure clinical and sub-clinical infection in the community, investigate socio-demographic and behavioural risk factors for influenza and generate novel data on antibody and T cell immunity, to inform influenza control initiatives.
- A total of 5484 participants were recruited from 2205 households randomly selected from registers of participating general practices.
- Participants were followed up for 118 158 person-weeks through six periods of influenza circulation: the winter seasons 2006/07, 2007/08 and2008/09, the summer 2009 pandemic wave, the winter 2009/10 pandemic wave and the post pandemic season 2010/11.
- The dataset comprises a wide range of demographic, social and behavioural measures, active weekly surveillance for respiratory illnesses and biological samples (nasal swabs, serology and T cells).
- Data are not currently open access but strategic collaborations are welcomed: enquiries to [a.hayward@ucl.ac.uk].

## Funding

This work is supported by awards establishing the Farr Institute of Health Informatics Research, London, from the MRC, in partnership with Arthritis Research UK, the British Heart Foundation, Cancer Research UK, the Economic and Social Research Council, the Engineering and Physical Sciences Research Council, the National Institute of Health Research, the National Institute for Social Care and Health Research (Welsh Assembly Government), the Chief Scientist Office (Scottish Government Health Directorates) and the Wellcome Trust (MR/K006584/1). O.D. is supported by a National Institute for Health Research Methods fellowship. M.S.C.L. was supported by a National Health and Medical Research Council Early Career Fellowship. Funding was also provided by the Medical Research Council and the Wellcome Trust (grant numbers: G0600511, G0800767 and MC_U122785833). The views expressed in this publication are those of the authors and not necessarily of the NHS, the NIHR or the Department of Health
